# Room Temperature Halide‐Eutectic Solid Electrolytes with Viscous Feature and Ultrahigh Ionic Conductivity

**DOI:** 10.1002/advs.202204633

**Published:** 2022-10-26

**Authors:** Ruonan Xu, Jingming Yao, Ziqi Zhang, Lin Li, Zhenyu Wang, Dawei Song, Xinlin Yan, Chuang Yu, Long Zhang

**Affiliations:** ^1^ Clean Nano Energy Center State Key Laboratory of Metastable Materials Science and Technology Yanshan University Qinhuangdao Hebei 066004 China; ^2^ Guilin Electrical Equipment Scientific Research Institute Co. Ltd. Guilin Guangxi 541004 China; ^3^ Tianjin Key Laboratory for Photoelectric Materials and Devices School of Materials Science and Engineering Tianjin University of Technology Tianjin 300384 China; ^4^ Institute of Solid State Physics Vienna University of Technology Wiedner Hauptstr. 8–10 Vienna 1040 Austria; ^5^ State Key Laboratory of Advanced Electromagnetic Engineering and Technology School of Electrical and Electronic Engineering Huazhong University of Science and Technology Wuhan Hubei 430000 China

**Keywords:** deep eutectic electrolytes, viscous solid electrolytes, ionic conductivity, halides, solid‐state batteries

## Abstract

A viscous feature is beneficial for a solid electrolyte with respect to assembling solid‐state batteries, which can change the solid‐solid contacts from point to face. Here, novel halide‐based deep eutectic solid electrolytes (DESEs) prepared by a facile ball milling method is reported. The mixture of halides triggers the deep eutectic phenomena by intermolecular interactions, leading to diverse morphologies and viscous statuses in terms of composition. Chemical‐ and micro‐structure analyses via the cryogenic technique reveal that the LiCl and LiF nanoparticles are dispersed in an amorphous halide matrix, which endow freely mobile ions for fast ion transport. The optimized DESE thus achieves low activation energy and high ionic conductivity of 16 mS cm^−1^ at room temperature, one of the highest values among various electrolytes so far. By integrating with the active materials to form a composite cathode, the viscous DESE yields a super‐dense composite pellet which possesses intensively enhanced ionic conductivity in contrast to those formed by the sulfide‐based electrolyte additives, demonstrating an attractive application prospect.

## Graphical abstract

Halide‐based deep eutectic solid electrolytes (DESEs) demonstrate impressively high ionic conductivity at room temperature by forming dispersive LiCl and LiF nanoparticles in the amorphous halide matrix that contains Li, Ga, Al, Cl, and F. The interaction of the dispersions and the matrix forms a Li‐ion solid electrolyte with interesting viscous features.

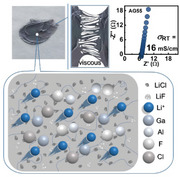

## 1 Introduction

The development of next‐generation electrochemical energy storage technology has raised urgent concerns with respect to high safety in recent years.^[^ ^1^ ^]^ Although the organic liquid electrolytes in conventional Li‐ion batteries have excellent electrode surface wettability and high ionic conductivity, they suffer basic problems that can cause security risks such as, flammability and leakage.^[^ ^2^ ^]^ One of the ways to solve these issues is to replace the liquid electrolyte by a solid electrolyte and use it to construct solid‐state batteries, where the solid parts can ensure good safety.^[^ ^3^ ^]^

Among diverse solid electrolytes, sulfides are particularly promising owing to their high ionic conductivity and relatively “soft” mechanical properties.^[^ ^4^ ^]^ However, during the solid‐state batteries assembling process, it is still hard to avoid the formation of pores in the electrolyte and electrodes due to the solid‐solid contacts. In addition, the thin and brittle ceramic electrolyte layer can be easily cracked.^[^ ^5^ ^]^ Furthermore, the detrimentally parasitic reactions of sulfide/oxide‐electrode and the poor solid‐solid contacts within the composite cathode can cause a large interfacial resistance, resulting in severe degradation of the electrochemical performance and battery failure.^[^ ^6^ ^]^

An effective approach to reduce the interface resistance is to introduce wetting agents at the interface such as, conductive flexible polymers, viscous gels, or ionic liquids with fluidity.^[^ ^7^ ^]^ The additives with good wettability can fill the defects and pores to enable intimate contacts between electrolyte and electrodes and reduce the interface resistance.^[^ ^8^ ^]^ Although the wetting agents provide a larger surface area for ion transport,^[^ ^9^ ^]^ the limited ionic conductivity of the additives drags down the overall ionic conductivity. The incorporation of the additives may accompany with organic solvents, which always react with and degrade the electrolytes. Moreover, many additives are unstable during electrochemical cycling and show poor mechanical properties.^[^ ^10^ ^]^

Unlike sulfides, the halide solid electrolytes show good compatibility toward the oxide‐ and phosphate‐based active materials and can construct a stable interface between them.^[^ ^11^ ^]^ Unfortunately, the ionic conductivity of the halides is relatively low. The halides also suffer the issue of poor solid‐solid contacts. Therefore, a halide solid electrolyte with high ionic conductivity and viscous characteristic is significant for realizing a low interface resistance, especially in the cathode.^[^ ^12^ ^]^

Deep eutectic electrolytes have attracted increasing attention because of their unique characteristics such as, structural flexibility as well as good thermal and chemical stability.^[^ ^13^ ^]^ The deep eutectic electrolytes are mixtures of molecules, ions, and/or molten salts,^[^ ^14^ ^]^ which may be synthesized in the absence of solvents. They are usually composed of two or more substances that interact with each other, and possess a lower melting point than their constituent substances.^[^ ^15^ ^]^ Generally, eutectic electrolytes are formed through three types of reactions named hydrogen bond interaction, Lewis acid‐base interaction, and Van der Waals interaction, which involve the complex interactions between anions and cations.^[^ ^14^, ^17^ ^]^ The eutectic electrolytes possess similar electrochemical properties and viscosities as ionic liquids, furthermore, they are cheaper and more environmentally friendly. Additionally, there is no further processing or purification procedure during the synthesis process of eutectic electrolytes, i.e., the utilization rate of raw materials is close to 100%.^[^ ^17^ ^]^ From these viewpoints, the development of deep eutectic solid electrolytes (DESEs) is particularly interesting.

Herein, we report a new type of viscous/flexible DESEs composed of LiCl, AlF_3_, and GaF_3_, prepared by a facile ball milling method. The intermolecular interaction between various components triggers a deep eutectic reaction. Different viscous states of DESEs are obtained by adjusting the proportion of the three components. The DESEs demonstrate the highest ionic conductivity of 16 mS cm^−1^ at room temperature, comparable to commercial liquid organic electrolytes. The diverse mechanical properties of DESEs with viscous/plastic features make them applicable in a variety of ways. The incorporation of the viscous DESEs in the LiFePO_4_ (LFP) active material powders enables solid‐solid face contacts and a pore‐ and crack‐free microstructure. A highly dense pellet can be produced that possesses intensively enhanced ionic conductivity compared with that mixed with sulfide‐based ion conductors. The as‐prepared eutectic composite can function not only as a solid electrolyte but also as an ion‐conductor additive used in electrodes.

## 2 Results and Discussion

**Figure 1:**
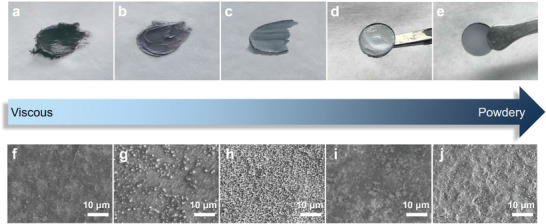
The formability of as‐prepared samples at room temperature. a–e) Optical photographs and f–j) SEM images for AG55 (a,f), AG64 (b,g), AG73 (c,h), AG82 (d,i), and AG91 (e,j), respectively.

The eutectic forming effect of the as‐prepared 2LiCl‐*x*AlF_3_‐(1 − *x*)GaF_3_ (0.5 ≤ *x* ≤ 0.9) DESEs changes with the ratio between AlF_3_ and GaF_3_. Video S1 (Supporting Information) displays different viscous/ductile behavior for the samples where *x* = 0.5, 0.6, 0.7, 0.8, and 0.9, which are designated as AG55, AG64, AG73, AG82, and AG91, respectively. The viscous feature changes significantly upon the ratio of AlF_3_/GaF_3_: The higher the proportion of GaF_3_, the stronger the interaction between anions and cations is, thereby the more obvious of the eutectic reaction. The optical photographs for different AlF_3_/GaF_3_ ratios are captured and shown in **Figure** **1**a–e. Both the AG55 and AG64 eutectic mixtures illustrate medium viscosity and stickness, which are easy to adhere to the preparing apparatus (Video S1, Supporting Information). With an increased AlF_3_/GaF_3_ ratio, AG73 exhibits plasticine‐like behavior, i.e., an increased plasticity for kneading. The good flexibility makes AG73 convenient to control its geometrical shape. AG82 is in an intermediate state between viscous and powdery, it thus shows partially agglomerated particles. After shaping in a mold, AG82 demonstrates a smooth surface. However, a further increase of the AlF_3_/GaF_3_ ratio in AG91 causes the loss of the viscous/ductile behavior, thereby AG91 presents a glass matrix in a powder state. This scenario indicates that a high AlF_3_ content recedes and finally vanishes the eutectic network.

From the SEM surface images (Figure 1f‐h), the 2LiCl‐*x*AlF_3_‐(1 − *x*)GaF_3_ DESEs demonstrate a highly dense structure without holes for *x* ≤ 0.7, with a morphology comparable to polymer‐based electrolytes.^[^ ^18^ ^]^ It is notable that the amount of granule precipitations intensively increases with increasing AlF_3_/GaF_3_ ratio. Up to AG91, the entire product consists of fine powders (Figure S1, Supporting Information), indicating a loss of deep eutectic phenomenon because of the insufficiency of GaF_3_. Nevertheless, the cold‐pressed AG82 and AG91 pellets remain more dense structures than sulfide‐based electrolytes,^[^ ^19^ ^]^ which can be also compared from the optical photograph (Figure 1d,e). Generally, with increasing *x* from 0.5 to 0.9, the composites change from a viscous to a powdery feature. The viscous features of AG55, AG64, AG73, and even AG82 DESEs enable them to act both as binders and as ion conductors in the electrode and/or electrolyte layers, which are favorable to: 1) enhance physical contacts between the active material powders and the additives, 2) eliminate pores in the electrolyte matrix beneficial for suppressing Li dendrite growth, and 3) improve ionic conductivity.

**Figure 2:**
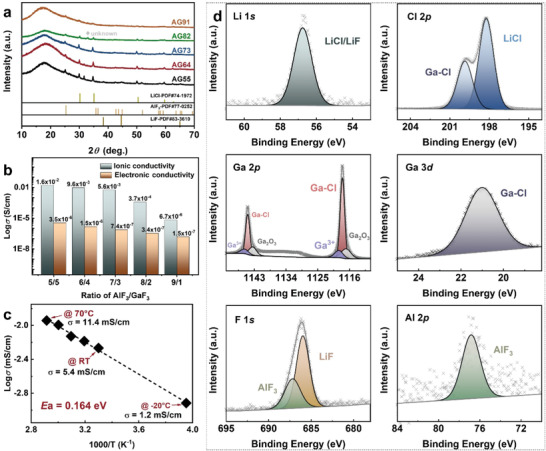
Physicochemical properties of 2LiCl‐*x*AlF_3_‐(1‐*x*)GaF_3_ (0.5 ≤ *x* ≤ 0.9). a) XRD patterns. b) Ionic and electronic conductivities. c) Arrhenius conductivity plot of AG73 measured from −25 to 70 °C. d) High‐resolution XPS spectra of AG55 for Li 1*s*, Cl 2*p*, Ga 2*p*, Ga 3*d*, F 1*s*, and Al 2*p*.

The XRD diffraction patterns of 2LiCl‐*x*AlF_3_‐(1‐*x*)GaF_3_ (0.5 ≤ *x* ≤ 0.9) are displayed in **Figure** **2**a. All samples show a composite of amorphous matrix mixed with crystal halides including LiCl, LiF, AlF_3_, and an unknown peak near 2*θ* = 32° (except for AG73). The enlarged LiF peak width indicates that LiF forms in nanoparticles. The major phase is in an amorphous state in this deep eutectic system.

Attributed to the amorphous and viscous features, the impedance spectra of the DESEs samples with 0.5 ≤ *x* ≤ 0.8 (Figure S2a‐d, Supporting Information) show almost vanished semicircles at the high and middle frequency regions, indicating a negligible grain boundary resistance. Considering that the bulk and grain boundary resistance of these samples cannot be distinguished, the total ionic conductivity was calculated from the local minimal resistance at the intersection of the impedance spectra. For the sample with *x* = 0.9 (Figure S2e, Supporting Information), the ionic conductivity was calculated from the fitting result with an equivalent circuit of *R*_b_(*R*_gb_‐CP*E*_gb_)W. As illustrated in Figure 2b, the ionic conductivity shows a downtrend with an increasing AlF_3_/GaF_3_ ratio. AG55 has the highest value of 16 mS cm^−1^ among as‐prepared samples, which is one of the highest ionic conductivity values so far.^[^ ^20^ ^]^ Even for AG73 with median ionic conductivity, the value (5.6 mS cm^−1^) is comparable to the high‐performance sulfide solid electrolytes.^[^ ^5^, ^22^ ^]^ However, AG91 in the powdery state only shows ionic conductivity as low as 6.7 × 10^−3^ mS cm^−1^. The electronic conductivity of the 2LiCl‐*x*AlF_3_‐(1‐*x*)GaF_3_ samples were also evaluated. The values (Figure 2b) decrease gradually with increasing AlF_3_/GaF_3_ ratio, indicating that the incorporation of AlF_3_ mitigates the electronic contribution. In all samples except for AG91, the electronic conductivity is more than three orders of magnitude lower than the ionic conductivity, confirming that the samples are pure ionic conductors.

The ionic conductivity is directly related to the deep eutectic formation ability of 2LiCl‐*x*AlF_3_‐(1‐*x*)GaF_3_. In this system, the higher degree of eutectic formation, the higher conductivity of the electrolyte. As listed in **Table** **1**, the DESEs with high ionic conductivity have remarkably viscous behavior. In contrast, low ionic conductivity comes from powdery samples. Importantly, the AlF_3_‐GaF_3_ collocation is the key factor to trigger the deep eutectic reaction. While at the same mole ratio, other components such as, the InF_3_‐GaF_3_, ZrCl_4_‐GaF_3_, Li_2_S‐GaF_3_, and AlF_3_‐GaCl_3_ collocations do not show deep eutectic phenomena, appearing the powdery state and low ionic conductivity. Albeit the ionic conductivity of LiCl/LiF is generally low, Li ions may interact with other components and can rapidly migrate across the eutectic phases.^[^ ^22^ ^]^ The effect of the ball milling time on ionic conductivity is also evaluated by prolonging the milling time from 6 to 12 h. As shown in Figure S3 (Supporting Information), the samples with a short milling time have slightly higher ionic conductivity than those with a long milling time. Therefore, 6 h is the optimal milling time for the eutectic reaction.

**Table 1:** Composition, physical state, and ionic conductivity (*σ*) of the as‐prepared samples

Composition	Physical state	*σ* (mS cm^−1^)
2LiCl‐0.5AlF_3_‐0.5GaF_3_	viscous (gel‐analogous)	16.0
2LiCl‐0.6AlF_3_‐0.4GaF_3_	viscous (gel‐analogous)	9.6
2LiCl‐0.7AlF_3_‐0.3GaF_3_	highly viscous (paste‐analogous)	5.4
2LiCl‐0.8AlF_3_‐0.2GaF_3_	negligibly viscous (clay‐analogous)	0.4
2LiCl‐0.9AlF_3_‐0.1GaF_3_	powdery	5.9 × 10^−3^
3LiCl‐0.3InF_3_‐0.7GaF_3_	highly viscous (paste‐analogous)	1.9
3LiCl‐0.5InF_3_‐0.5GaF_3_	negligibly viscous (clay‐analogous)	0.2
3LiCl‐0.7InF_3_‐0.3GaF_3_	powdery	7.6 × 10^−4^
2LiCl‐0.5AlF_3_‐0.5GaCl_3_	powdery	6.3 × 10^−3^
AlF_3_‐GaCl_3_	powdery	−
2LiCl‐0.5ZrCl_4_‐0.5GaF_3_	powdery	1.3 × 10^−3^
LiCl‐Li_2_S‐GaF_3_	powdery	1.1 × 10^−2^

Considering the good flexibility (Video S1, Supporting Information) and the well geometrical shape controllability, AG73 was selected to measure the activation energy *E*_a_. Figure 2c shows the temperature‐dependent ionic conductivity in the range of −20–70 °C. The data follow a good linear relationship, indicating the good phase stability in the DESE composite over the measured temperature range. The ionic conductivity of AG73 remains 1.2 mS cm^−1^ at the lowest temperature of −20 °C (Figure S4, Supporting Information) and 11.4 mS cm^−1^ at the highest temperature of 70 °C. Thus, the as‐prepared DESEs can be used in a broad temperature range, with the assumption that AG55 and AG64 should have higher ionic conductivity than AG73. By fitting the linear Arrhenius plot, the activation energy of AG73 was determined to be *E*_a_ = 0.164 eV. Compared with typical sulfide solid electrolytes (e.g., Li_6_PS_5_Cl, *E*_a_ = 0.333 eV) and halide solid electrolytes (e.g., Li_3_InCl_6_, *E*_a_ = 0.326 eV),^[^ ^23^ ^]^ the activation energy of AG73 is significantly lower, indicating much faster transport kinetics.

XPS analyses were carried out with AG55, which possesses the highest ionic conductivity, to evaluate the resulting compositions from the eutectic reaction. The obtained high‐resolution XPS spectra are depicted in Figure 2d. In the binding energy (BE) range of the Li 1*s* spectrum, the peak is attributed to the overlaps of LiCl (56.7 eV) and LiF (56.8 eV), which are too close to be distinguished.^[^ ^24^ ^]^ The peak position of Cl 2*p* at 199.8 eV (the lower peak) is related to Ga‐Cl compounds such as, CaCl, GaCl_2_, and/or GaCl_3_.^[^ ^25^ ^]^ The other peak (the higher peak) at 198.2 eV is related to LiCl, albeit it slightly deviates from the peak position of the standard LiCl (198.9 eV).^[^ ^26^ ^]^ We thus speculate that LiCl in AG55 is embedded in an amorphous halide matrix. The mixture has thus the interaction between the complex anion and cation that causes the shift of the LiCl peak. For the Ga 2*p* spectrum, the core energy level value of Ga has a positive displacement relative to an elemental Ga (1116.5–1116.7 eV),^[^ ^27^ ^]^ which indicates that the Ga atom in the

electrolyte is a compound state.^[^ ^28^ ^]^ The two peaks at 1118.0 and 1144.9 eV with the highest intensity can be attributed to Ga–Cl,^[^ ^29^ ^]^ which is further confirmed from the peak at 21.0 eV in the Ga 3*d* spectrum.^[^ ^30^ ^]^ The satellite peaks at 1119.0 and 1146.2 eV are assigned to Ga^3+^ in the electrolyte.^[^ ^31^ ^]^ The peaks at 1117.0 and 1143.5 eV in Ga 2*p* attributed to Ga_2_O_3_ could be induced by oxidation during the XPS sample preparation.^[^ ^32^ ^]^ The peak positions of F 1*s* at 685.9 and 687.2 eV are respectively attributed to the presence of LiF and AlF_3_ in the composite.^[^ ^33^ ^]^ For the Al 2*p* spectrum, the peak appears at 76.8 eV which is attributed to AlF_3_.^[^ ^34^ ^]^ These results confirm that the deep eutectic reaction forms a halide composite with LiCl, LiF, AlF_3_, and Ga–Cl compounds.

**Figure 3:**
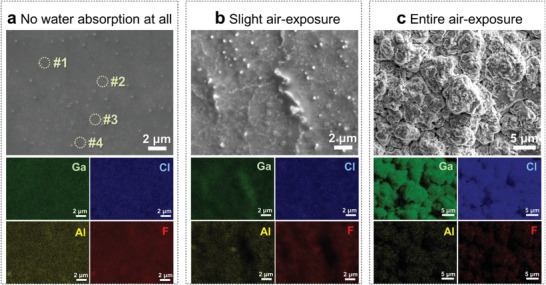
SEM detections on AG55 with different degrees of air‐exposures. a) Absolute air‐isolation. b) Slight air‐exposure. c) Entire air‐exposure.

The composition of AG55 was investigated using SEM‐EDS. The morphology of AG55 (**Figure** **3**a) shows nanoparticles dispersed in the amorphous matrix. The EDS point detection data on various locations (Table S1, Supporting Information) demonstrate similar elemental mole ratios, indicating a homogeneous composite. The mole percentages of halogens (F and Cl) are in the range of 55%–75%. During the sample transfer, we found the composite is highly hygroscopic. Some places on the sample surface have obviously changed by air exposure, as shown in Video S2 (Supporting Information). Interestingly, the nanoparticles are expanded after reacting with the absorbed moisture and burst the matrix layer, so a compositional analysis for AG55 was further performed through different degrees of moisture exposures. Under a slight water absorption (Figure 3b), the nanoparticles are expanded and connected to form a stripe shape that bulges out the matrix. The EDS mapping shows that the stripe shape region is Ga‐rich and no Al or F is detectable. The morphology changes to a cauliflower shape (Figure 3c) after an entire air exposure. Ga and Cl elements are conspicuous in the corresponding elemental mapping. This is because Cl‐based compounds are hygroscopic, the absorbed water can migrate to the inner of the matrix and then reacts with GaCl_2_ which intensively changes the microstructure. An EDS point detection (#1 in Figure S5 and Table S2, Supporting Information) shows that this area is Ga–Cl rich. Along with the XPS data (Figure 2d), we propose that this area is composed of GaCl_2_, which is extremely hygroscopic. In contrast, the ratio of Al, F, and Cl elements in the amorphous matrix area is similar to the dry sample (Figure 3a). Although the presence of moisture has a certain effect on the microstructure, it does not change the viscous status (Video S3, Supporting Information) of the AG55 composite, nor the macro‐appearance, as shown in Figure S6 (Supporting Information). The ionic conductivity of AG73 as a function of air‐exposure time (room temperature, ≈40% humidity) is shown in Figure S7 (Supporting Information). The value of the pristine sample is 5.9 mS cm^−1^, comparable to that of the AG73 sample shown in Figure 2b. With prolonging the air‐exposure time, the ionic conductivity increases (up to 13 mS cm^−1^ after 10 min exposure time). This is because the presence of LiCl·*x*H_2_O leads to an increase of ionic conductivity.^[^ ^35^ ^]^

**Figure 4:**
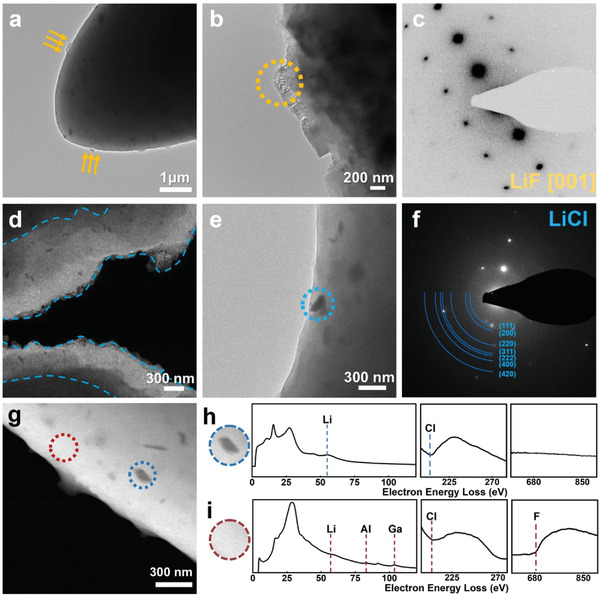
Cryo‐TEM characterization of AG55. a) TEM bright field image at the edge of AG55. b) Selected area on panel a (marked with arrows) with a high magnification. c) SAED obtained from the rough area on panel b (marked with a dotted circle). d) STEM image under the annular dark field model. e) Nanoparticles embedded in the amorphous matrix and, f) its SAED. g‐i) EELS detections on the embedded particle (h) and the matrix (i). The dashed lines on the panels are guides to the eye.

To evaluate the amorphous matrix constituent, EDS combined with an EELS analysis was performed on the AG55 sample using cryo‐TEM, as shown in **Figure** **4**. The samples for TEM were held at −170 °C during the testing process to avoid damages by the electron beam and the moisture. From the magnified edge layer (yellow arrow marked in Figure 4a), some rough areas embedded in the matrix are clearly seen (Figure 4b). The selected area electron diffraction (SAED, Figure 4c) reveals a LiF diffraction pattern on these rough areas. Similar rough areas also can be observed on other locations (Figure S8, Supporting Information). With the annular dark field model (Figure 4d), granular substances are found to be randomly distributed inside the amorphous matrix. After a relatively big particle (Figure 4e) has been irradiated with the electron beam, the amorphous matrix coated on the particle can be removed. The SAED analysis on the particle shows a typical LiCl diffraction pattern, as shown in Figure 4f. The LiCl particles with a dark contrast are scattered in the matrix, which may act as an ionic conductive phase in the DESE.^[^ ^36^ ^]^

STEM‐EELS measurements were performed on embedded particles and the matrix, as shown in Figure 4g, circled regions. In particles, a disordered LiCl (Figure 4h) is obtained by the low loss EELS, consistent with the SAED result. The scattered LiCl and/or LiF in the DESE could be another reason beneficial for the ultrafast Li‐ion transport.^[^ ^37^ ^]^ In contrast, the amorphous matrix (Figure 4i) illustrates a significantly different profile, where the characteristic peaks of Li–K edge, Al–L edge, Ga–M edge, Cl–L edge, and F–K edge are all detected. Additionally, the STEM‐HAADF EDS mapping (Figure S9, Supporting Information) confirms a homogeneous elemental distribution on the amorphous matrix. According to previous studies, a deep eutectic phenomenon can occur when the intermolecular interactions of different components are stronger than the original components of an individual substance.^[^ ^38^ ^]^ The properties of DESEs are strongly related to the intermolecular interactions between different components.^[^ ^13a^ ^]^ The presence of freely moving ions enables the high conductive ability in the DESE.^[^ ^39^ ^]^ Therefore, combined with SEM/STEM EDS, XPS, and EELS, we propose that the matrix is composed of various halides, which interact with each other to form a deep eutectic composite with a viscous feature and ultrahigh ionic conductivity.

**Figure 5:**
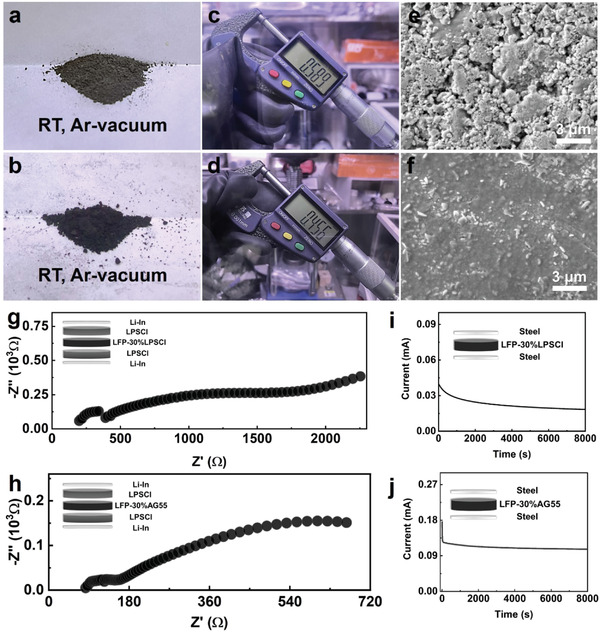
Evaluations on the mixture of LFP and ion conductor additives (LPSCl or AG55). The powders of a) LFP‐30%LPSCl and b) LFP‐30%AG55. The thickness of cold‐pressed c) LFP‐30%LPSCl and d) LFP‐30%AG55 pellets and e‐f) their SEM surface morph morphology. g‐h) Impedance spectrum and i–j) DC polarization curve of LFP‐30%LPSCl (g,i) and LFP‐30%AG55 (h,j).

The chemical stability of AG55 to Li metal was investigated, as shown in Figure S10 (Supporting Information). After contacting AG55 with the Li metal, the surface color of Li metal turns black in 1 h, indicating that the as‐prepared 2LiCl‐*x*AlF_3_‐(1‐*x*)GaF_3_ electrolytes are chemically unstable to the Li metal, similar to other halide electrolytes reported previously.^[^ ^40^ ^]^ However, owing to the merits of viscosity and ductility, DESEs can be used as a binder in the cathode to bond the active material and the carbon additive, and to eliminate the pores between the particles of the active material. Meanwhile, viscous DESEs can act as an ion conductor in the cathode (this is necessary for all‐solid‐state batteries), and coat on the active material to improve the physical contact and to realize face‐to‐face ion transports, rather than point‐to‐point ion transports observed in traditional solid‐solid contacts regarding sulfide and oxide solid electrolytes. As an example, a sample with 30 wt.% AG55 electrolyte mixed with 70 wt.% LFP active material (denoted as LFP‐30%AG55) was compared with the one with 30 wt.% Li_6_PS_5_Cl electrolyte mixed with LFP (denoted as LFP‐30%LPSCl), as shown in **Figure** **5**. Different from the LFP‐30%LPSCl (Figure 5a) and pure LFP (Figure S11a, Supporting Information) powders, the LFP‐30%AG55 powders (Figure 5b) were glued to big particles. That is, the LFP‐30%LPSCl and pure LFP powders remain a loose structure while the LFP‐30%AG55 powders agglomerate together by AG55 due to its high viscosity. After powder cold pressing, the pellets of these composites show different thicknesses. The thickest pellet is LFP‐30%LPSCl (0.589 mm, Figure 5c), while the thinnest pellet is LFP‐30%AG55 (0.456 mm, Figure 5d) and that of the pure LFP baseline (0.516 mm) is intermediate (Figure S11b, Supporting Information). Compared with the SEM surface morphology, the LFP‐30%AG55 pellet (Figure 5f) demonstrates a very dense microstructure without pores or cracks. In contrast, a mass of pores exists in the LFP‐30%LPSCl (Figure 5e) and pure LFP (Figure S11c, Supporting Information) pellets. This scenario confirms that the presence of viscous AG55 can well bind LFP powders and significantly densifies the pellet, while the sulfide‐based ionic additives cannot contribute to the densification.

The ionic and electronic conductivity of the LFP‐additive mixtures were measured on symmetric cells to evaluate the different conducting effects of the LPSCl and AG55 additives. Considering Li–In is a mixed conductor, to strictly block the electron conduction, two LPSCl solid electrolyte pellets were used as ion conduction and electron insulation layers in the symmetric cells to study the ionic conductivity,^[^ ^41^ ^]^ as shown in Figure 5g,h. Li–In foils were used as electrodes to maintain a stable LPSCl/Li‐In interface.^[^ ^42^ ^]^ The impedance spectra show that the resistance of LFP‐30%AG55 (Figure 5h) is much smaller than that of LFP‐30%LPSCl (Figure 5g). The cell with the pure LFP (Figure S11d, Supporting Information) has the highest impedance. For the study of electron charge transfer (Figure 5i,j), stainless steel foils were used as ion insulation and electron conduction electrodes. The electronic conductivity of LFP‐30%AG55 (9.7 × 10^−6^ S cm^−1^) is higher than that of LFP‐30%LPSCl (4.2 × 10^−6^ S cm^−1^). This may be understood by the denser structure in LFP‐30%AG55 owing to the viscous feature of AG55 and the higher electronic conductivity of AG55 in contrast to LPSCl. However, due to the relative electron insulation feature of AG55 and LPSCl, their incorporations into LFP reduce the electronic conductivity about an order of magnitude in comparison to pure LFP (Figure S11e, Supporting Information). This shortcoming can be overcome by adding carbon additives. Consequently, the incorporation of viscous DESEs can improve the ion transport performance in the composite cathode, by changing from rigid point‐contacts to soft face‐contacts and improving the densification of the composite cathode. Moreover, the viscous DESEs may act as a buffer zone favorable to mitigate the volume expansion in the cathode during electrochemical cycling.

## 3 Conclusion

In conclusion, the 2LiCl‐*x*AlF_3_‐(1‐*x*)GaF_3_ (0.5 ≤ *x* ≤ 0.9) deep eutectic composite system composed of halides shows viscous/flexible characteristic and ultrahigh ionic conductivity in a broad temperature range. The AlF_3_‐GaF_3_ collocation is the key factor to trigger the deep eutectic reaction, which is directly related to the Li‐ion transport. The higher the GaF_3_ concentration, the deeper the eutectic reaction and thereby the higher ionic conductivity can be. Typically, AG55 possesses a high ionic conductivity of 16 mS cm^−1^ at room temperature. Combined with the diverse evaluations including Cryo‐STEM‐EELS, LiCl and LiF nanoparticles were found to be dispersed in a Li‐Al‐Ga‐based halides amorphous matrix, which constitute the DESEs. The LiCl/LiF dispersions in the matrix are also responsible for the ultrafast Li‐ion transport in the DESE, because the Li ions can interact with the eutectic phases to enable a rapid migration. AG55‐incorporated LFP‐30%AG55 can be compacted into a highly dense pellet without any pores or cracks, achieving the thinnest pellet, compared to the pellets compacted with pure LFP and LFP‐30%LPSCl. Moreover, the ionic conductivity of the LFP‐based cathode is intensively enhanced by AG55‐incorporation. Consequently, DESEs can be used not only as good solid electrolytes, but also as additives in the electrodes with the merits: to improve the ion transport performance of electrodes, to realize soft face‐contacts between active materials and additives, and to act as a buffer zone to mitigate the volume expansion during electrochemical cycling.

## 4 Experimental Section

### Synthesis of the DESEs

DESEs were prepared by high‐energy ball milling. The LiCl (aladdin, 99.9%), GaF_3_ (J&K Chemicals, 99.99%), and AlF_3_ (aladdin, 99.99%) precursors were weighted and preliminarily mixed in a agate mortar based on stoichiometric proportions of 2LiCl‐*x*AlF_3_‐(1 − *x*)GaF_3_ (0.5 ≤ *x* ≤ 0.9)), which were designated as AG55 (*x* = 0.5), AG64 (*x* = 0.6), AG73 (*x* = 0.7), AG82 (*x* = 0.8), and AG91 (*x* = 0.9). Ball milling was performed on a Fritsch P7 premium apparatus with ZrO_2_ vials and balls (10 mm) at 500 rpm for 6 and 12 h, respectively. The commercial materials are: Li_6_PS_5_Cl (LPSCl, GLESI China), Li–In alloy (thickness 100 µm, Hawk China), LiFePO_4_ (LFP, MTI China). All purchased materials were used as‐received without any treatment.

### Preparation of the LFP‐Additive Mixtures

The LFP‐additive mixtures were prepared through mixing LFP with LPSCl or AG55 additive in a weight ratio of 70:30, which is respectively designated as LFP‐30%LPSCl or LFP‐30%AG55. The weighted powders were first hand‐ground in an agate mortar for 10 min, and then ball‐milled (Fritsch P7 premium) with ZrO_2_ vials and balls (10 mm) at 200 rpm for 1 h. The resulting mixtures and the baseline LFP were respectively cold‐pressed into pellets at ≈350 MPa.

### Material Characterization

X‐ray diffraction (XRD) were performed on a Rigaku D/MAX‐2500/PC (Cu K*α*) in a 2*θ* range of 10–70° with a scan rate of 3° per min. The X‐ray photoelectron spectroscopy (XPS) analyses were collected using Al K*α* microfocus monochromatic source under a low powder of 72 W. The detailed spectra were recorded using CAE scanning mode with a pass energy from 30 to 50 eV. The morphological analyses were carried out on an SEM (Hitachi S‐4800 II FESEM) equipped with EDS. Selected area electron diffraction (SAED) and electron energy loss spectroscopy (EELS) analyses were performed using a Cs‐corrected environmental transmission electron microscope (ETEM, Titan G2, Thermo Fisher Scientific). High‐angle annular dark‐field scanning transmission electron microscopy (STEM‐HAADF) and corresponding EDS were performed in a TEM (Talos F20, Thermo Fisher Scientific) at 200 kV. The SEM and TEM samples were transferred in an Ar‐protected holder.^[^ ^41b^ ^]^ TEM was measured at a cryogenic temperature of −170 °C.

Electrochemical impedance spectroscopy (EIS) was carried out on an impedance analyzer (Princeton 4000) within the frequency range of 1 Hz to 3 MHz and a bias of 0.2 V. The detailed symmetric cell structures are shown in the main text. Li–In foils were used as electrodes. Ionic and electronic conductivities were measured on steel|SE|steel symmetric cells. For preparing the SE pellets for AG55, AG64, and AG73 with viscous features, the samples were pasted in between the steel foils and pressed into a die at ≈2 MPa. For the AG82 and AG91 powders, the pellets were cold‐pressed at 350 MPa. The pellets are 8 mm in diameter. Electronic conductivity was tested according to the Wagner's polarization method using Princeton 4000 by applying a constant voltage of 0.5 V. The bias was held for 8000 s. A Faraday cage was used during the measurement.

## Conflict of Interest

The authors declare no conflict of interest.

## Supporting information

Supporting Information

Supplemental Video 1

Supplemental Video 2

Supplemental Video 3

## Data Availability

The data that support the findings of this study are available from the corresponding author upon reasonable request.
