# Supplementary material for: Room Temperature Halide‐Eutectic Solid Electrolytes with Viscous Feature and Ultrahigh Ionic Conductivity
Source: Adv Sci (Weinh). 2022 Oct 26;9(35):2204633. doi: 10.1002/advs.202204633 (PMC9762297; doi:10.1002/advs.202204633)
Supplement: Supplementary file 1 — Supporting Information [file ADVS-9-2204633-s004.pdf]

ADVANCED SCIENCE
Open Access

Supporting Information

for *Adv. Sci.*, DOI 10.1002/advs.202204633

Room Temperature Halide-Eutectic Solid Electrolytes with Viscous Feature and Ultrahigh Ionic Conductivity

*Ruonan Xu, Jingming Yao, Ziqi Zhang, Lin Li, Zhenyu Wang, Dawei Song, Xinlin Yan, Chuang Yu and Long Zhang**

Copyright WILEY-VCH Verlag GmbH & Co. KGaA, 69469 Weinheim, Germany, 2022.

Supporting Information

Room Temperature Halide-Eutectic Solid Electrolytes with Viscous Feature and Ultrahigh Ionic Conductivity

Ruonan Xu[1], Jingming Yao[1], Ziqi Zhang[1], Lin Li[1], Zhenyu Wang[2], Dawei Song[3], Xinlin Yan[4], Chuang Yu[5], and Long Zhang[1,*]

[1]Clean Nano Energy Center, State Key Laboratory of Metastable Materials Science and Technology, Yanshan University, Qinhuangdao 066004, Hebei, China

[2]Guilin Electrical Equipment Scientific Research Institute Co. Ltd., Guilin, 541004, Guangxi, China

[3]Tianjin Key Laboratory for Photoelectric Materials and Devices, School of Materials Science and Engineering, Tianjin University of Technology, Tianjin 300384, China

[4]Institute of Solid State Physics, Vienna University of Technology, Wiedner Hauptstr. 8-10, 1040 Vienna, Austria

[5]State Key Laboratory of Advanced Electromagnetic Engineering and Technology, School of Electrical and Electronic Engineering, Huazhong University of Science and Technology, Wuhan 430000, Hubei, China

*Corresponding author E-mail: lzhang@ysu.edu.cn

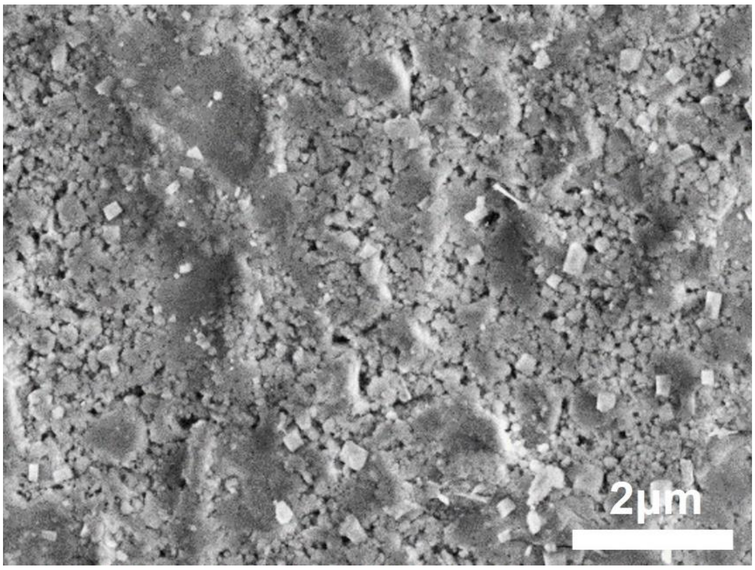

Figure S1. SEM image of AG91 with a higher magnification.

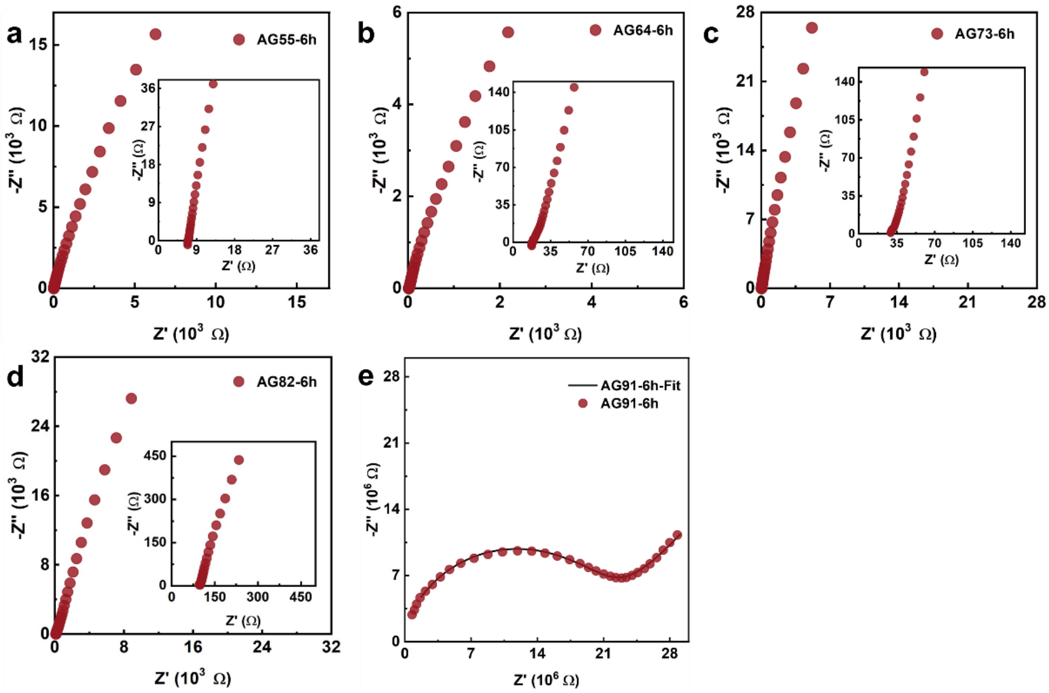

Figure S2. EIS Nyquist plots of 2LiCl-xAlF$_3$-(1-x)GaF$_3$ ($0.5 \leq x \leq 0.9$) prepared with ball milling time of 6h. a) AG55. b) AG64. c) AG73. d) AG82. e) AG91.

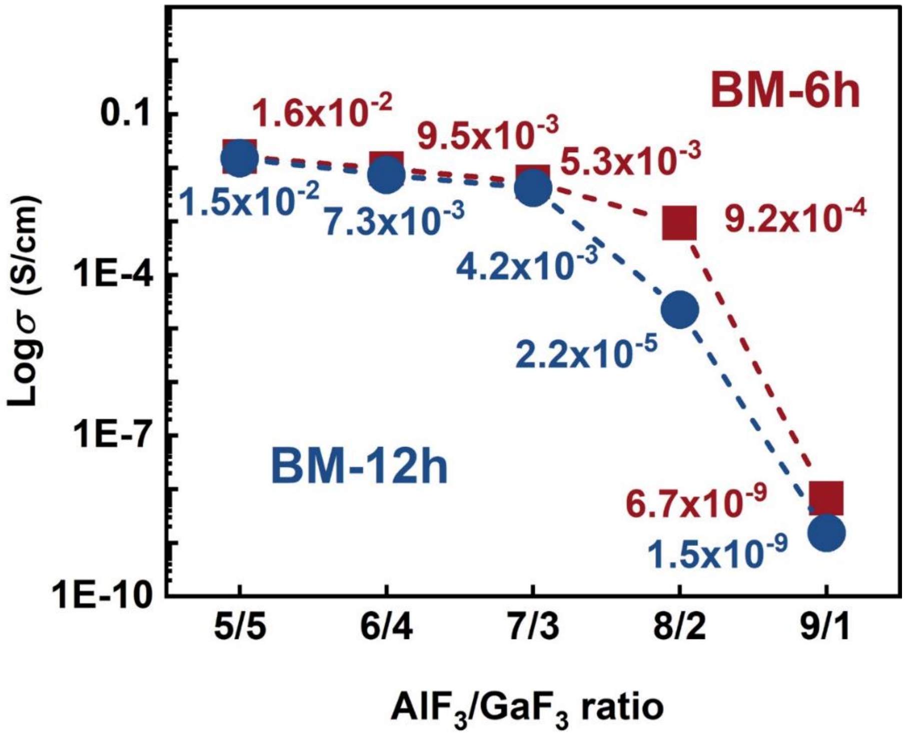

Figure S3. Ionic conductivity of 2LiCl-xAlF$_3$-(1-x)GaF$_3$ ($0.5 \leq x \leq 0.9$) prepared with different ball milling times (6 and 12 h).

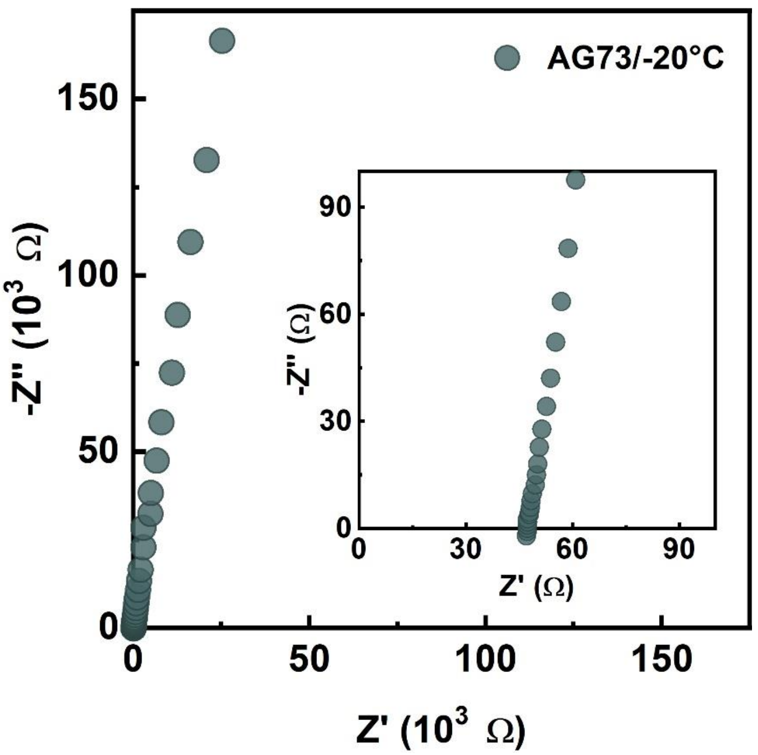

Figure S4. EIS Nyquist plot of AG73 at -20 °C.

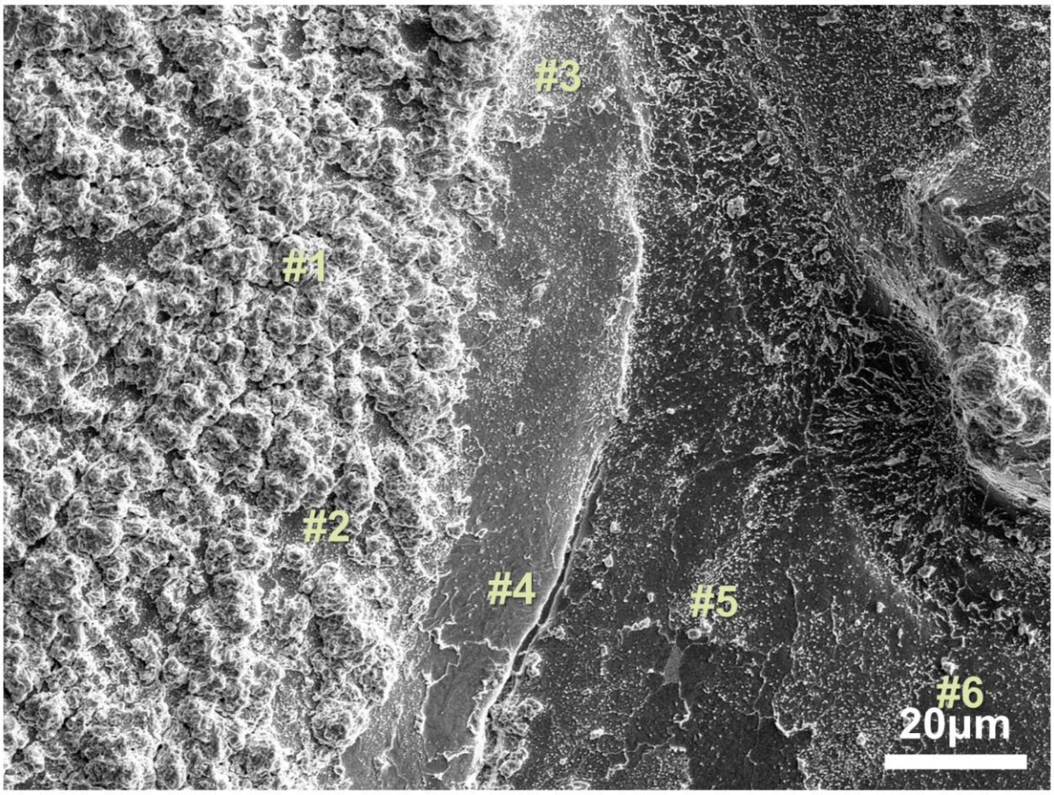

Figure S5. SEM image of AG55 after air-exposure. The EDS point analyses were performed on the selected spots from #1 to #6. The corresponding EDS elemental data are listed in Table S2.

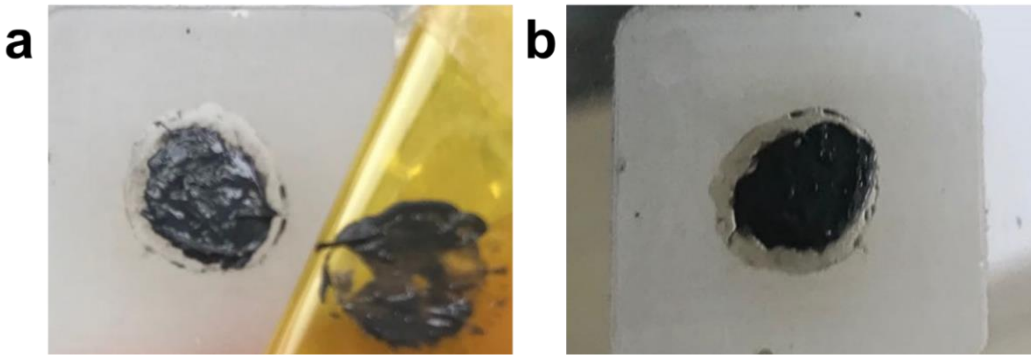

Figure S6. Aqueous sensitivity test of AG55. a) Pristine state. b) Exposed in air for 30 min.

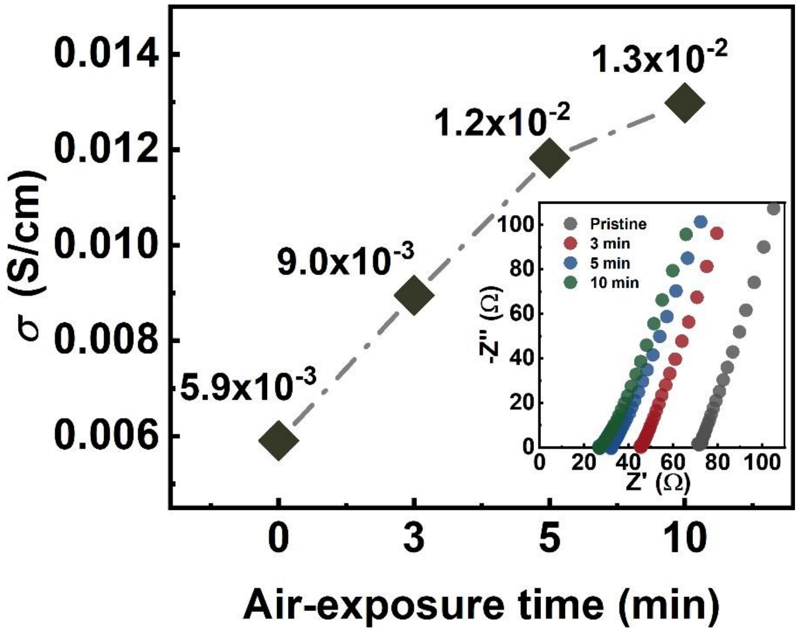

Figure S7. Ionic conductivity of AG73 as a function of air-exposure time.

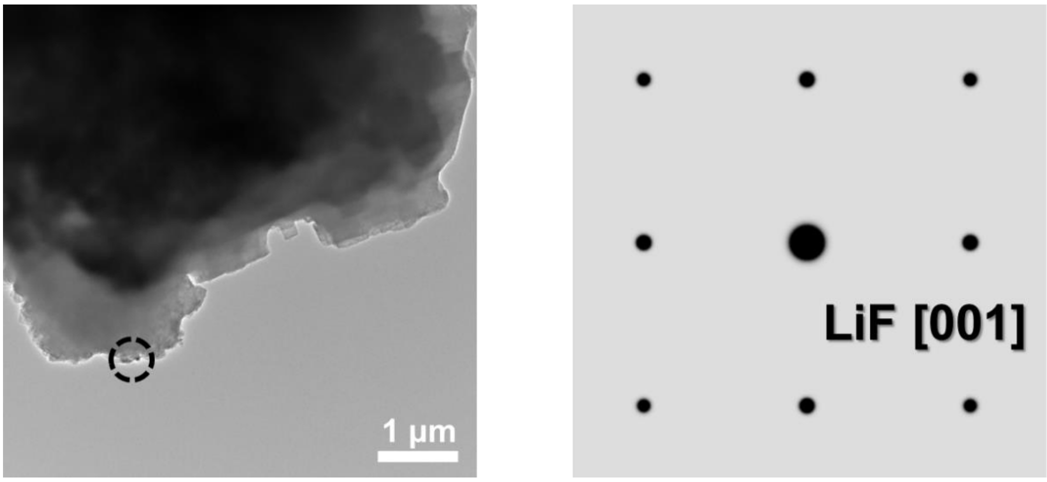

Figure S8. Cryo-TEM measurement. The selected rough area (left panel) and the corresponding SAED pattern (right panel).

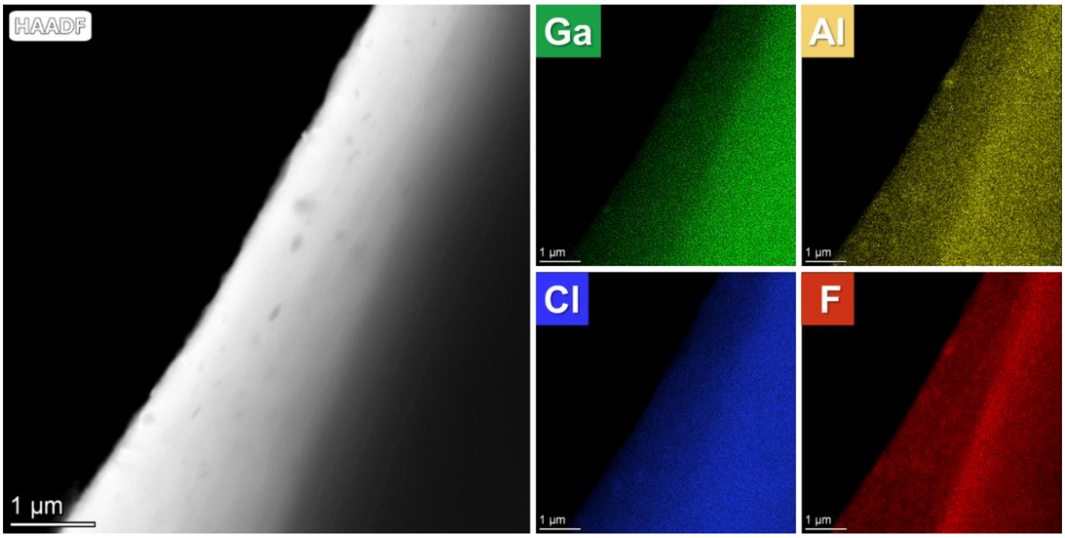

Figure S9. STEM-HAADF and EDS mapping images for AG55.

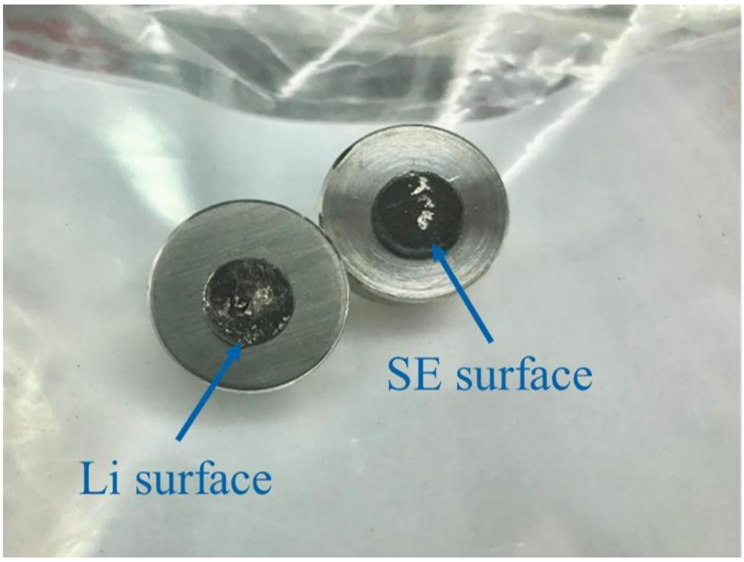

Figure S10. Chemical stability of AG73 toward Li metal. The surface of the Li metal turns black after contacting with AG73 for 1 h.

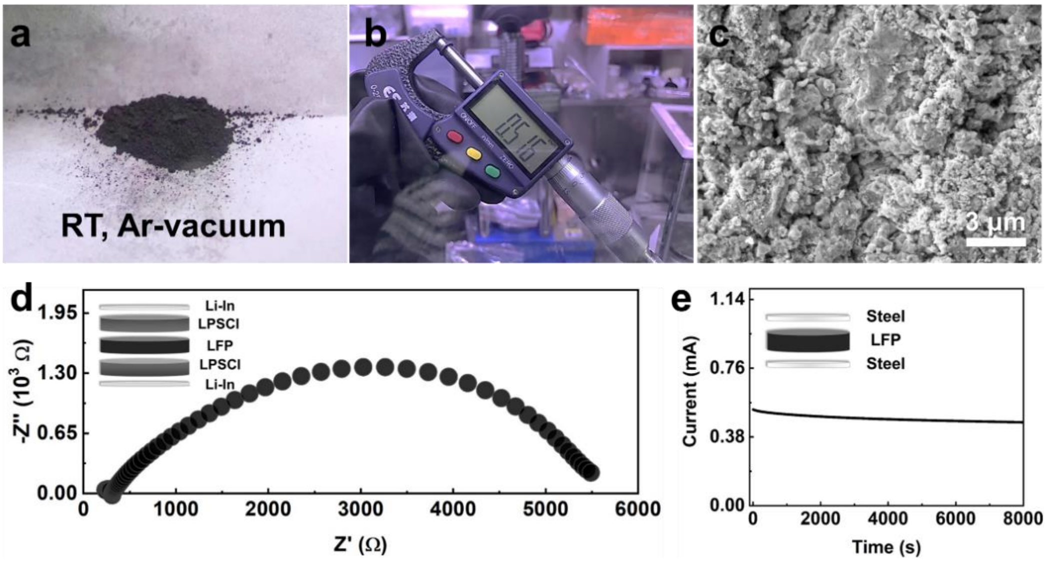

Figure S11. Evaluations on LFP. a) Optical photograph of the powders. b) Thickness of the pellet cold-pressed from the powders (60 mg). c) SEM image of the pellet. d) Nyquist plot of the pellet. e) DC polarization curve of the pellet.

Table S1. EDS point analysis of AG55 shown in Figure 3a.

Spot	Ga	F	Al	Cl
1	8.64	33.92	8.11	40.44
2	7.58	33.83	7.70	40.37
3	7.02	38.35	8.15	38.22
4	5.30	28.90	6.72	26.33

Table S2. EDS point analysis of air-exposed AG55 shown in Figure S4.

Spot	Ga	F	Al	Cl
1	75.27	0.00	1	128.94
2	1.28	2.78	1	3.94
3	1.31	3.78	1	3.41
4	1.31	3.33	1	3.60
5	1.18	2.26	1	5.34
6	0.89	1.74	1	3.87
